# Comparison of young Japanese underweight women with eating disorder tendencies and constitutional thinness: A cross-sectional study

**DOI:** 10.1016/j.pmedr.2025.103186

**Published:** 2025-07-22

**Authors:** Mariko Ogawa, Michiko Nakazato, Jinko Yokota, Kaori Koga

**Affiliations:** a Fukushima Medical Center for Children and Women, Fukushima Medical University, 1, Hikarigaoka, Fukushima City, Fukushima 960-1295, Japan; b Department of Psychiatry, International University of Health and Welfare, School of Medicine, 4-3, Kozunomori, Narita City, Chiba 286-8686, Japan; c Health Care Center, Tokyo Women's Medical University, 8-1, Kawada-Cho, Shinjuku-Ku, Tokyo 162-8666, Japan; d Department of Obstetrics and Gynecology, Reproductive Medicine, Graduate School of Medicine, Chiba University, 1-8-1 Inohana Chuo-ku Chiba City, Chiba 260-8670, Japan

**Keywords:** Anemia, Body dissatisfaction, Eating disorders, Female, Young adult

## Abstract

**Objective:**

To compare eating disorder tendencies and constitutional thinness in young underweight Japanese women and identify distinguishing factors.

**Methods:**

In September 2024, 1000 young Japanese women were recruited and categorized into three groups based on body mass index (BMI) and responses to the Sick, Control, One Stone, Fat, and Food (SCOFF) questionnaire: eating disorder tendency (BMI <18.5 kg/m^2^ and SCOFF-positive, *n* = 93), constitutional thinness (BMI <18.5 kg/m^2^ and SCOFF-negative, *n* = 219), and control (BMI 18.5–25.0 kg/m^2^ and SCOFF-negative, *n* = 435).

**Result:**

The eating disorder tendency group idealized a thinner body shape than the constitutional thinness group and reported greater dissatisfaction with their body shape; however, body dissatisfaction was highest in the control group. General health habits were similar between the constitutional thinness and control groups. Among underweight women, significant predictors of eating disorder tendency included history of anemia (odds ratio [OR]: 4.27; 95 % confidence interval [CI]: 2.13–8.56), daily physical activity (OR: 3.46; 95 % CI: 1.78–6.74), and eating before bedtime (OR: 2.44; 95 % CI: 1.16–5.15).

**Conclusions:**

General health habits differ between underweight women with and without eating disorder tendencies. A history of anemia may serve as a potential indicator for screening for eating disorders.

## Highlights

• Compared underweight women with/without eating disorder tendencies.
• Underweight women with/without eating disorder tendencies differed in lifestyle.
• Anemia history predicted eating disorder tendencies in underweight women.
• Routine screening should assess eating disorder risk, including anemia history.

## 1 Introduction

Eating disorders are psychiatric conditions that are often difficult to treat. For instance, anorexia nervosa, characterized by remarkable thinness, has a high mortality rate (Treasure et al., 2020). According to the Diagnostic and Statistical Manual of Mental Disorders, Fifth Edition, Text Revision (DSM-5-TR), diagnosis requires a significantly lower body weight, with severity indicated by the body mass index (BMI) (American Psychiatric Association, 2022). However, not all individuals with a low BMI can be classified as having an eating disorder.

Constitutional thinness refers to a severe state of persistent underweight without disordered eating behaviors (Bailly et al., 2020). Unlike anorexia nervosa, constitutional thinness is typically associated with normal menstrual function and normal levels of female and thyroid hormones (Estour et al., 2017). Although bone quality is impaired in both conditions (Fernández-García et al., 2009), the underlying mechanisms differ. In anorexia nervosa, osteoporosis management focuses on weight restoration (National Institute for Health and Care Excellence (NICE), 2020), whereas individuals with constitutional thinness generally maintain normal fat-free mass, which is even higher than in those with anorexia nervosa (Bailly et al., 2021a). Consequently, the benefit of fat supplementation in constitutional thinness remains unclear. Despite these clinical distinctions, research on constitutional thinness in Japanese women remains limited.

The diagnostic criteria for anorexia nervosa have changed since the introduction of the DSM-5, such as eliminating amenorrhea (American Psychiatric Association, 2022), making this distinction more difficult. In Japan, approximately 20 % of women in their 20s and 30s have been underweight for over a decade (Ministry of Health, Labour and Welfare, 2023). Studies suggest that Asian women tend to have lower BMI and body fat percentages than their Caucasian counterparts (Conroy et al., 2011), influenced by genetic (Hur et al., 2008) and cultural factors (Olson et al., 2020). Furthermore, Asian women exhibit lower leptin and adiponectin levels at equivalent BMIs (Conroy et al., 2011), and a tendency toward higher body fat percentage and abdominal obesity—factors associated with increased insulin resistance risk (Wulan et al., 2010). These findings highlight the need to consider cultural and ethnic backgrounds when evaluating body composition and eating behaviors.

Understanding differences between underweight women with and without eating disorder tendencies may support early identification and appropriate care. Women with eating disorder tendencies require clinical assessment and intervention, whereas constitutionally thin women should be informed about the potential risk of bone loss. Therefore, we aimed to focus on the differences in the general health habits of young underweight Japanese women and examine the differences between underweight women with and without eating disorder tendencies.

## 2 Materials and methods

### 2.1 Participants and study design

**Fig. 1:**
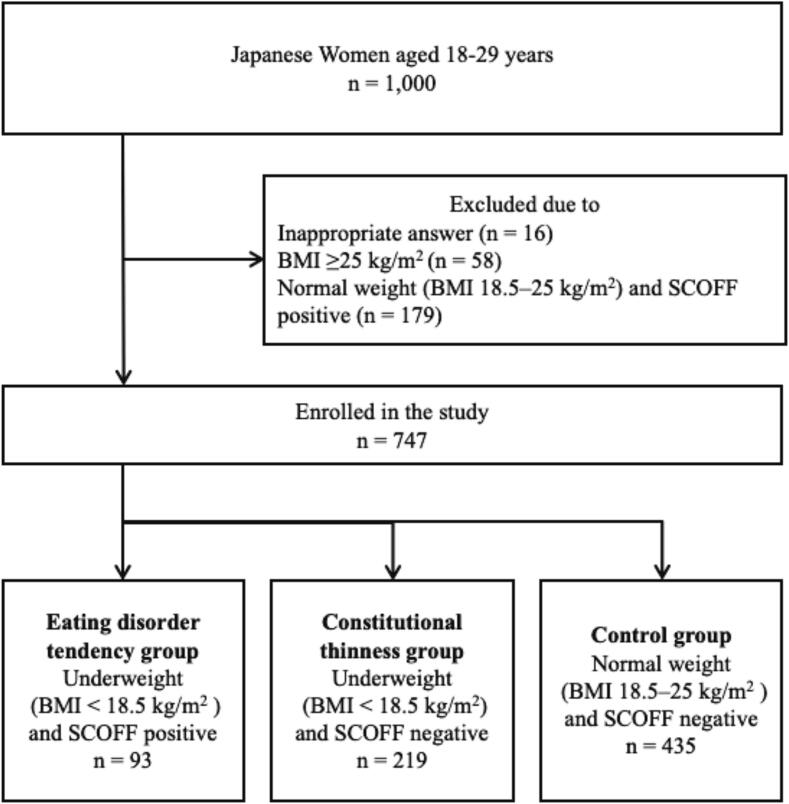
Flowchart of participant selection among young Japanese women in 2024. Note: SCOFF-positive was defined as a score of two or more, indicating eating disorder tendencies. SCOFF-negative was defined as a score of less than two.

A cross-sectional survey was conducted among young Japanese women. A total of 1000 women aged 18 to 29, evenly distributed across age groups, participated in a web-based questionnaire conducted on September 26–27, 2024. Participants were randomly selected from a web panel (Cross Marketing Inc., Japan). All participants were informed of the study's purpose and gave consent before completing the questionnaire, which included items related to body shape and eating habits. A flow diagram of participant enrollment is presented in Fig. 1. Underweight was defined as BMI <18.5 kg/m^2^, and normal weight as a BMI of 18.5–25.0 kg/m^2^, according to guidelines from the National Heart, Lung, and Blood Institute (Am. J. Clin. Nutr., 1998) and the Japanese Society for the Study of Obesity (Kanazawa et al., 2005).

Participants were categorized into three groups based on their BMI and responses to the Sick, Control, One Stone, Fat, and Food (SCOFF) questionnaire. A score of two or more indicated SCOFF-positive status (high risk of eating disorders), and a score of less than two indicated SCOFF-negative status (Hill et al., 2010):

(1) Eating disorder tendency group: underweight and SCOFF-positive.

(2) Constitutional thinness group: underweight and SCOFF-negative.

(3) Control group: normal weight and SCOFF-negative.

Inclusion criteria were: Japanese women living and educated in Japan, appropriate responses to height and weight questions, and either a BMI <18.5 kg/m^2^ or a BMI of 18.5–25.0 kg/m^2^ with SCOFF-negative status. Exclusion criteria included a history of childbirth, which may influence body image (Lovering et al., 2018), invalid height or weight data, BMI of 18.5–25.0 kg/m^2^ with SCOFF-positive status, and a BMI ≥25.0 kg/m^2^.

Background characteristics, menstrual status, body image and satisfaction, general health and lifestyle habits, and factors influencing ideal body shape were compared among the three groups.

### 2.2 Survey items

The survey items were developed through discussions among two gynecologists, a psychiatrist, and an internist.

#### 2.2.1 SCOFF questionnaire

We used the five-item SCOFF questionnaire to assess the risk of eating disorders. The SCOFF is designed for use by non-specialists in primary care and has demonstrated good sensitivity for detecting anorexia nervosa and bulimia nervosa (Hill et al., 2010; Kutz et al., 2020). The Japanese version has been validated and is widely used (Hosoda et al., 2021). Two or more positive responses suggest a possible eating disorder and warrant further evaluation.

#### 2.2.2 Counter drawing rating scale

The Counter Drawing Rating Scale (CDRS) (Thompson and Gray, 1995) assesses body dissatisfaction using figure drawings. Participants selected figures representing (1) their perceived body shape and (2) their ideal body shape from nine silhouettes ranging from very underweight to obese. Discrepancy scores range from −8 to −1 for those desiring a thinner shape, and from 1 to 8 for those preferring a larger shape (Dion et al., 2015). The CDRS has demonstrated reliability and validity (Wertheim et al., 2004).

#### 2.2.3 General health and lifestyle habits

To understand the general health and lifestyle habits of the participants, the necessary items were extracted from the General Health Examination Questionnaire developed by the Japanese Ministry of Health, Labour and Welfare (Ministry of Health, Labour and Welfare, 2020). It is widely used in Japan for general health checkups. We selected questions regarding history of anemia, smoking history, physical activity, dietary habits, and alcohol consumption from the questionnaire. The item on weight change was excluded because of overlap with an existing SCOFF question.

### 2.3 Statistical analyses

BMI was calculated as weight (kg) divided by height squared (m^2^). Weight and height were self-reported. Data on participant characteristics were reported using means, standard deviations, and 95 % confidence intervals (CIs) for continuous variables and proportions for categorical variables. Categorical variables were analyzed using the chi-squared test with Bonferroni-adjusted post hoc comparisons. Continuous variables that violated the assumption of homogeneity of variance were analyzed using Welch's ANOVA, followed by Games–Howell post hoc tests. Statistical significance was defined as *P* < 0.05.

A binary logistic regression model was used to assess the association between independent variables and eating disorder tendencies in underweight women. Independent variables included age and responses to a general health examination questionnaire. The outcome variables were dichotomized as follows: SCOFF-positive = 1 and SCOFF-negative = 0. The odds ratios (ORs) with 95 % CIs were calculated. The goodness-of-fit of the model was evaluated using the Hosmer–Lemeshow test (*P* = 0.84).

A *P*-value of 0.05 was considered statistically significant. All data were analyzed using the SPSS software (version 29.0; IBM Corp., Armonk, NY, USA).

### 2.4 Ethics

This study was conducted in accordance with the principles of the Declaration of Helsinki. The Ethics Committee of Fukushima Medical University determined that ethical review was not required for this study (REC2024–094), as it involved anonymized data and posed minimal risk to participants.

## 3 Results

### 3.1 Comparison of the background characteristics and the menstrual cycle

The background characteristics of the participants in the entire group and each group are shown in Table 1. No significant differences were observed between the three groups regarding marital status, occupation, or educational level. The eating disorder tendency group was significantly younger than the control group (*P* = 0.04). Menstrual irregularities (30.6 %) and amenorrhea (12.5 %) were more prevalent in the eating disorder tendency group compared to the control group (17.3 % and 0.8 %, respectively). The constitutional thinness group had a lower incidence of amenorrhea (3.6 %) than the eating disorder tendency group but a higher rate than the control group. Regarding changes in appetite before menstruation, fewer respondents in the eating disorder tendency group answered “never” compared to the other two groups. There were no significant differences between the constitutional thinness and control groups in this regard.

**Table 1:** Comparison of background characteristics in 2024 among underweight young Japanese women with and without eating disorder tendencies, and normal-weight women without eating disorder tendencies.

	All (*n* = 747)	Underweight		Control (n = 435)	*P*-value
		Eating disorder tendency (*n* = 93)	Constitutional thinness (*n* = 219)		
	Mean (SD) or n (%)	Mean (SD) or n (%)	Mean (SD) or n (%)	Mean (SD) or n (%)	
Age (years)	23.6 (3.5)	22.9^a^ (3.5)	23.5^a,b^ (3.4)	23.9^b^ (3.4)	0.04

Marital status					
Single	674 (90.2)	87 (93.5)	193 (88.1)	394 (90.6)	0.31
Married	73 (9.8)	6 (6.5)	26 (11.9)	41 (9.4)	
Living situation					
Alone	234 (31.3)	29 (31.2)	61 (27.9)	144 (33.1)	0.66
With guardians	400 (53.5)	53 (57.0)	125 (57.1)	222 (51.0)	
With a partner	94 (12.6)	8 (8.6)	28 (12.8)	58 (13.3)	
Others	19 (2.5)	3 (3.2)	5 (2.3)	11 (2.5)	

Occupation					
Student	213 (28.5)	35 (37.6)	63 (28.8)	115 (26.4)	0.18
Worker	423 (56.6)	44 (47.3)	127 (58.0)	252 (57.9)	
Housewife	10 (1.3)	1 (1.1)	3 (1.4)	6 (1.4)	
Unemployed	80 (10.7)	13 (14.0)	22 (10.0)	45 (10.3)	
Others	21 (2.8)	0 (0.0)	4 (1.8)	17 (3.9)	

Education level					
< High school graduate	45 (6.0)	7 (7.5)	12 (5.5)	26 (6.0)	0.98
High school graduate	208 (27.8)	26 (28.0)	61 (27.9)	121 (27.8)	
> High school graduate	494 (66.1)	60 (64.5)	146 (66.7)	288 (66.2)	

Income ($/year)					
<13,300	433 (58.0)	61 (65.6)	131 (59.8)	241 (55.4)	0.57
13,300–40,000	290 (38.8)	29 (31.2)	82 (37.4)	179 (41.1)	
≥40,000	24 (3.2)	3 (3.2)	6 (2.7)	15 (3.4)	

Hormone therapy use					
No	598 (80.1)	70^a^ (75.3)	184^a^ (84.0)	344^a^ (79.1)	0.03
Yes	108 (14.5)	21^a^ (22.6)	27^a^ (12.3)	60^a^ (13.8)	
Do not know	41 (5.5)	2^a^ (2.2)	8^a^ (3.7)	31^a^ (7.1)	

Menstrual cycle†					
Regular	397 (62.1)	36^a^ (50.0)	122^a^ (63.5)	239^a^ (63.7)	<0.01
Irregular	126 (19.7)	22^a^ (30.6)	39^a,b^ (20.3)	65^b^ (17.3)	
Amenorrhea	19 (3.0)	9^a^ (12.5)	7^b^ (3.6)	3^c^ (0.8)	
Do not know	97 (15.2)	5^a^ (6.9)	24^a^ (12.5)	68^a^ (18.1)	

Changes in appetite before menstruation					
Yes, each premenstrual period	194 (26.0)	33^a^ (35.5)	51^a^ (23.3)	110^a^ (25.3)	0.04
Yes, but not every time	286 (38.3)	39^a^ (41.9)	88^a^ (40.2)	159^a^ (36.6)	
Never	267 (35.7)	21^a^ (22.6)	80^b^ (36.5)	166^b^ (38.2)	

Note: Group definitions: “Eating disorder tendency” = underweight with a positive result on an eating disorder screening; “Constitutional thinness” = underweight with a negative screening result; “Control” = normal weight with a negative screening result.

For continuous variables, the mean (SD) was calculated using Welch's ANOVA, followed by Games–Howell post hoc tests. For categorical variables (%), *P*-values were calculated using the chi-squared test with Bonferroni-adjusted post hoc comparisons.

Superscript letters (^a^, ^b^, ^c^) indicate differences between variables or proportions at the same grading level among the three groups. If all values within a variable or proportion are marked with the same superscript letter (e.g., “^a^”), it indicates no significant differences between groups at that level. Different superscript letters (e.g., “^a^” vs. “^b^”) indicate significant differences between the marked values. $ = U.S. dollars. Income converted from Japanese yen (1 USD ≈ 150 JPY).

† Excludes participants undergoing hormone therapy (*n* = 108).

### 3.2 Comparison of the subjective ideal and healthy BMI, body image, and body dissatisfaction

Table 2 shows a comparison of subjective ideal and healthy BMI, body image, body dissatisfaction, and experience with weight loss behaviors among the three groups. Although actual BMI did not significantly differ between the eating disorder tendency and constitutional thinness groups, subjective ideal and healthy BMIs were lower in the eating disorder tendency group than in the constitutional thinness group (subjective ideal: 16.6 ± 1.5 kg/m^2^ vs. 17.4 ± 1.7 kg/m^2^; subjective healthy: 17.8 ± 2.0 kg/m^2^ vs. 18.3 ± 1.5 kg/m^2^, respectively). The control group had higher values for subjective ideal and healthy BMI compared to the other two groups. The eating disorder tendency group had more participants who recognized their body shapes as slightly overweight (11.8 %) than the constitutional thinness group (1.4 %). Additionally, 35.5 % of the eating disorder tendency group reported dissatisfaction with their body shape, compared to 18.3 % in the constitutional thinness group. However, there was no significant difference in body shape satisfaction between the eating disorder tendency and control groups. On the CDRS, the eating disorder tendency group selected a thinner ideal body shape compared to the other two groups, whereas the constitutional thinness and control groups had similar preferences. Despite this, the control group reported the highest body dissatisfaction scores (1.5 ± 1.4). The eating disorder tendency group had a significantly higher dissatisfaction score (0.7 ± 1.9) than the constitutional thinness group (−0.1 ± 1.4). Participants in the eating disorder tendency group were also more likely to have attempted weight loss in the past. Current weight loss behaviors were less common in the constitutional thinness group compared to the eating disorder tendency and control groups.

**Table 2:** Comparison of body image, body satisfaction, and weight loss behaviors in 2024 among underweight young Japanese women with and without eating disorder tendencies, and normal-weight women without eating disorder tendencies.

	Underweight		Control (*n* = 435)	P-value
	Eating disorder tendency (*n* = 93)	Constitutional thinness (n = 219)		
	mean (SD) or n (%)	mean (SD) or n (%)	mean (SD) or n (%)	
Actual BMI (kg/m^2^)	16.8^a^ (1.5)	17.0^a^ (1.7)	20.5^b^ (1.5)	<0.01
Subjective ideal BMI (kg/m^2^)	16.6^a^ (1.9)	17.4^b^ (1.7)	18.96^c^ (1.5)	<0.01
Subjective healthy BMI (kg/m^2^)	17.8^a^ (2.0)	18.3^b^ (1.5)	19.60^c^ (1.6)	<0.01

What do you think of your body shape?				
Underweight	25^a^ (26.9)	67^a^ (30.6)	9^b^ (2.1)	<0.01
Slightly underweight	29^a^ (31.2)	83^a^ (37.9)	50^b^ (11.5)	
Normal	25^a^ (26.9)	65^a^ (29.7)	249^b^ (57.2)	
Slightly overweight	11^a^ (11.8)	3^b^ (1.4)	106^c^ (24.4)	
Overweight	3^a, b^ (3.2)	1^b^ (0.5)	21^a^ (4.8)	

Are you satisfied with your body shape?				
Satisfied	14^a, b^ (15.1)	52^b^ (23.7)	67^a^ (15.4)	<0.01
Somewhat dissatisfied	37^a^ (39.8)	90^a^ (41.1)	182^a^ (41.8)	
Dissatisfied	33^a^ (35.5)	40^b^ (18.3)	123^a^ (28.3)	
Can't say either way	9^a^ (9.7)	37^a^ (16.9)	63^a^ (14.5)	

Contour Drawing Rating Scale†				
Perceived body shape	2.9^a^ (1.6)	2.6^a^ (1.2)	4.4^b^ (1.4)	<0.01
Ideal body shape	2.1^a^ (1.2)	2.7^b^ (1.3)	2.9^b^ (1.3)	<0.01
Body dissatisfaction	0.7^a^ (1.9)	-0.1^b^ (1.4)	1.5^c^ (1.4)	<0.01

Weight loss behaviors				
Past	71^a^ (76.3)	79^b^ (36.1)	262^c^ (60.2)	<0.01
Current	51^a^ (54.8)	48^b^ (21.9)	188^a^ (43.2)	<0.01

Note: Group definitions: “Eating disorder tendency” = underweight with a positive result on an eating disorder screening; “Constitutional thinness” = underweight with a negative screening result; “Control” = normal weight with a negative screening result.

For continuous variables, the mean (SD) was calculated using Welch's ANOVA, followed by Games–Howell post hoc tests. For categorical variables (%), P-values were calculated using the chi-squared test with Bonferroni-adjusted post hoc comparisons.

Superscript letters (^a^, ^b^, ^c^) indicate differences between variables or proportions at the same grading level among the three groups. If all values within a variable or proportion are marked with the same superscript letter (e.g., “^a^”), it indicates no significant differences between groups at that level. Different superscript letters (e.g., “^a^” vs. “^b^”) indicate significant differences between the marked values.

† The greater values indicate greater body dissatisfaction.

### 3.3 Comparison of the general health habits and factors predicting SCOFF positivity

Table 3 shows a comparison of general health habits between the groups. The eating disorder tendency group had a significantly higher prevalence of a history of diagnosed anemia (39.8 % vs. 14.6 % in the constitutional thinness group and 15.9 % in the control group). They were also more likely to engage in daily physical activity (51.6 % vs. 30.1 %, 32.4 %), eat before going to bed (35.5 % vs. 16.0 %, 16.3 %), and consume sweets between meals (31.2 % vs. 18.7 %, 15.2 %). There were no significant differences between the constitutional thinness and control groups across any of these items.

**Table 3:** Comparison of general health habits in 2024 among underweight young Japanese women with and without eating disorder tendencies, and normal-weight women without eating disorder tendencies.

	Underweight		Control (n = 435)	P-value
	eating disorder tendency (n = 93)	Constitutional thinness (n = 219)		
	n (%)	n (%)	n (%)	
Have you ever been diagnosed as anemic?	37^a^ (39.8)	32^b^ (14.6)	69^b^ (15.9)	<0.01
Are you currently a heavy smoker? ^†^	6 (8.2)	8 (4.3)	13 (3.6)	0.20
Are you in a habit of doing exercise to sweat lightly for over 30 min a time, 2 times weekly, for over a year?	21 (22.6)	32 (14.6)	111 (14.9)	0.08
In your daily life, do you walk or do any equivalent amount of physical activity for more than 1 h a day?	48^a^ (51.6)	66^b^ (30.1)	141^b^ (32.4)	<0.01
Is your walking speed faster than the speed of those of your age and sex?	37 (39.8)	90 (41.1)	161 (37.0)	0.58
Is your eating speed quicker than others?	30 (32.3)	51 (23.3)	111 (25.5)	0.25
Do you eat supper 2 h before bedtime more than 3 times a week?	33^a^ (35.5)	35^b^ (16.0)	71^b^ (16.3)	<0.01
Do you eat snacks or drink sweet beverages between meals?	29^a^ (31.2)	41^b^ (18.7)	66^b^ (15.2)	<0.01
Do you skip breakfast more than 3 times a week?	37^a^ (39.8)	64^ab^ (29.2)	99^b^ (22.8)	0.02
How often do you drink? (sake, shochu, beer, wine, whisky, or brandy, etc.) †				
Everyday	4 (5.5)	7 (3.7)	22 (6.0)	0.68
Sometimes	29 (39.7)	64 (34.2)	124 (34.1)	
Rarely drink	40 (54.8)	116 (62.0)	218 (59.9)	

Note: Group definitions: “Eating disorder tendency” = underweight with a positive result on an eating disorder screening; “Constitutional thinness” = underweight with a negative screening result; “Control” = normal weight with a negative screening result.

The P-value was calculated using the chi–squared test with Bonferroni-adjusted post hoc comparisons.

Superscript letters (^a^, ^b^) indicate differences between proportions at the same grading level among the three groups. If all proportions are marked with the same superscript letter (e.g., “^a^”), it indicates no significant differences at that level. Different letters (e.g., “^a^” vs. “^b^”) indicate significant differences between the marked proportions.

† Eating disorder tendency (*n* = 73), constitutional thinness (*n* = 187), and control (*n* = 364). This question was asked of those who were older than 20 years of age.

The results of the binary logistic regression analyzing general health habits that predicted eating disorder tendency in underweight women are shown in Table 4. Factors significantly predictive of eating disorder tendency included history of anemia (OR = 4.27; 95 % CI: 2.13–8.56), daily physical activity (OR = 3.46; 95 % CI: 1.78–6.74), and eating before going to bed (OR = 2.44; 95 % CI: 1.16–5.15).

**Table 4:** Binary logistic regression predicting eating disorder tendencies in young underweight Japanese women based on general health habit factors in 2024.

	Adjusted Odds Ratio	95 % CI
Have you ever been diagnosed as anemic?	4.27	2.13, 8.56
Are you currently a heavy smoker?	2.41	0.64, 9.01
Are you in a habit of doing exercise to sweat lightly for over 30 min a time, 2 times weekly, for over a year?	0.76	0.32, 1.79
In your daily life, do you walk or do any equivalent amount of physical activity for more than 1 h a day?	3.46	1.78, 6.74
Is your walking speed faster than the speed of those of your age and sex?	0.66	0.35, 1.26
Is your eating speed quicker than others?	1.78	0.89, 3.59
Do you eat supper 2 h before bedtime more than 3 times a week?	2.44	1.16, 5.15
Do you eat snacks or drink sweet beverages between meals?	1.33	0.64, 2.76
Do you skip breakfast more than 3 times a week?	1.03	0.54, 1.99
How often do you drink? (sake, shochu, beer, wine, whisky, or brandy, etc.)	0.89	0.50, 1.56

Note: Age was adjusted for in the analysis.

### 3.4 Comparison of the most influential factors in achieving the ideal body shape

When asked about the factors that most influenced their body shape (Table 5), the most common response in the eating disorder tendency group was the body shape of famous people, followed by information on social media, and the body shape and opinions of friends. In the constitutional thinness group, the most common answer was “nothing in particular,” followed by the body shape of famous people and “do not know.” In the control group, the most frequent response was also the body shape of famous people, followed by “nothing in particular” and “do not know.” The percentage of respondents selecting “nothing in particular” was lower in the eating disorder tendency group than in the other two groups. Conversely, the percentage choosing “the body shape and opinions of friends” was higher in the eating disorder tendency group than in the control group.

**Table 5:** Most influential factors for achieving the ideal body shape in 2024 among underweight young Japanese women with and without eating disorder tendencies, and normal-weight women without eating disorder tendencies.

	Underweight				Control (n = 435)		*P*-value
	eating disorder tendency (n = 93)		Constitutional thinness (n = 219)				
	n	%	n	%	n	%	
Learned at school	10^a^	10.8	30^a^	13.7	49^a^	11.3	<0.01
Body shape of famous people	22^a^	23.7	33^a^	15.1	85^a^	18.7	
Body shape/opinions of friends	13^a^	14	14^a, b^	6.4	26^b^	7.1	
Opinions of a partner	5^a^	5.4	4^a^	1.8	9^a^	2.4	
Opinions of parents	5^a^	5.4	8^a^	3.7	11^a^	3.2	
Information from TV, magazines, internet	2^a^	2.2	13^a^	5.9	28^a^	5.8	
Social media	16^a^	17.2	28^a^	12.8	53^a^	13	
Medical professionals	4^a^	4.3	7^a^	3.2	6^a^	2.3	
Nothing in particular	6^a^	6.5	47^b^	21.5	76^b^	17.3	
Do not know	10^a^	10.8	32^a^	14.6	86^a^	17.1	
Others	0^a^	0	3^a^	1.4	6^a^	1.2	

Note: Group definitions: “Eating disorder tendency” = underweight with a positive result on an eating disorder screening; “Constitutional thinness” = underweight with a negative screening result; “Control” = normal weight with a negative screening result.

The P-value was calculated using the chi–squared test with Bonferroni-adjusted post hoc comparisons.

Superscript letters (^a^, ^b^) indicate differences between proportions at the same grading level among the three groups. If all proportions are marked with the same superscript letter (e.g., “^a^”), it indicates no significant differences at that level. Different letters (e.g., “^a^” vs. “^b^”) indicate significant differences between the marked proportions.

## 4 Discussion

This study explored differences between underweight women with and without eating disorder tendencies, focusing on general health habits.

Irregular menstruation and amenorrhea were more prevalent in the eating disorder tendency group than in the control group in this study. Amenorrhea is common in anorexia nervosa and is caused by hypothalamic–pituitary–ovarian axis dysfunction as an adaptation to a low-energy state resulting from starvation (Schorr and Miller, 2017). However, women with constitutional thinness do not have irregular menstruation, and the levels of their sex hormones, such as estradiol, are normal (Bailly et al., 2021b). The results of our study support these findings and suggest that it is important to check for menstrual irregularities and body weight when screening patients for eating disorders.

In this study, although actual BMIs were similar, participants with eating disorder tendencies perceived lower subjective ideal and healthy body weights than those with constitutional thinness. They were more dissatisfied with their body shape. One study reported that patients with anorexia nervosa perceived their actual body shape as fatter and showed greater restraint in eating than women with constitutional thinness (Bang et al., 2020). A study conducted in Japan indicated a positive correlation between being underweight, aspiration for thinness, experiences related to weight management, and eating disorders (Mase et al., 2013). Our findings support these results. However, the SCOFF questionnaire used for screening includes an item asking whether respondents consider themselves overweight, which may have influenced the outcomes.

Interestingly, normal-weight women without eating disorder tendencies reported greater body dissatisfaction than underweight women with such tendencies. Young Japanese women face strong societal pressure to attain a thinner body ideal, shaped by complex sociocultural factors. Women's media often promote the “Cinderella weight,” corresponding to a BMI of 18.0 kg/m^2^ (Iizuka et al., 2023). Therefore, it is unsurprising that many normal-weight women express dissatisfaction when measured against such ideals.

In addition to daily physical activity and eating before going to bed, a history of anemia was a significant predictor of eating disorder tendency in underweight women in this study. Although exercise and eating before bed are known to be associated with disordered eating, no previous study has reported that a history of anemia distinguishes constitutional thinness from eating disorders. Anemia is common in anorexia nervosa, with a meta-analysis reporting a prevalence of 44.8 % (Salari et al., 2025). The causes include bone marrow suppression, nutritional deficiencies, hemolysis, and inflammation (Mamou et al., 2016). In contrast, one study found no differences in red blood cell parameters between individuals with anorexia nervosa and those with constitutional thinness (Bang et al., 2020). Although we did not perform blood tests in this study, our findings suggest that inquiring about a history of anemia, along with dietary habits, may help distinguish eating disorders from constitutional thinness.

In this study, the most influential factor affecting ideal body shape in the eating disorder tendency group was the body shape of famous people, whereas “nothing in particular” was the most common in the constitutional thinness group. The eating disorder tendency group had fewer respondents selecting “nothing in particular” and more citing “the body shape and opinions of friends.” These findings suggest that women with eating disorder tendencies are more likely to compare their body shape with others and to be influenced by media than those with constitutional thinness or a healthy weight. A meta-analysis found that exposure to media images promoting thinness is associated with body image concerns in women (Grabe et al., 2008). Social media use has also been identified as a risk factor for eating disorders (Dane and Bhatia, 2023). Increased exposure to appearance-focused content and triggering material may contribute to the development of eating disorders, beyond individual vulnerability. Media has been reported as a significant source of appearance-related pressure among young Japanese women (Ando et al., 2021). In contrast, research on the influence of body image in constitutional thinness remains scarce. Our findings suggest that women with constitutional thinness may be less influenced by media regarding physical appearance compared to those with eating disorder tendencies.

This study has certain limitations. First, this study was conducted among women registered with an online survey company, which may limit the generalizability of the findings. Notably, 31.2 % of participants were underweight—higher than the national estimate of approximately 20 %—suggesting the sample may differ from the general population. Second, all variables—including height, weight, lifestyle habits, and SCOFF responses—were self-reported, introducing potential recall bias or inaccuracies. Young women often overestimate height and underestimate weight, particularly in the context of eating psychopathology (Meyer et al., 2009). Ideally, actual measurements should be used. Patients with anorexia nervosa may also underestimate physical activity (Bezzina et al., 2019), and self-administered questionnaires may overestimate the prevalence of mental disorders compared to diagnostic interviews (Zimmerman, 2024). Moreover, a positive SCOFF result does not confirm the presence of an eating disorder or indicate its specific type. Although useful for screening anorexia and bulimia nervosa, the SCOFF does not detect conditions like avoidant/restrictive food intake disorder (ARFID), which can cause severe weight loss without body image disturbance (American Psychiatric Association, 2022). Therefore, some participants classified as constitutionally thin may have had ARFID or other undiagnosed disorders. Future research should compare underweight women with and without a formal diagnosis by a psychiatrist. Although individuals with a childbirth history were excluded, this may have inadvertently removed a specific biological subgroup, potentially affecting representativeness. Despite these limitations, this is, to our knowledge, the first study to examine differences between eating disorder tendencies and constitutional thinness in a general sample of young Japanese women. We also identified predictors of eating disorder tendencies using general health examination items. As patients with eating disorders often visit primary care clinics, these findings highlight the need to equip primary care providers to distinguish eating disorder tendencies from constitutional thinness in underweight women. Further studies are needed to develop effective screening methods.

## 5 Conclusion

Among young underweight Japanese women, a history of anemia—along with disordered eating behaviors—was identified as a predictor of eating disorder tendencies. In primary care, assessing anemia history may help screen for eating disorders.

## CRediT authorship contribution statement

**Mariko Ogawa:** Writing – original draft, Project administration, Methodology, Investigation, Formal analysis, Data curation, Conceptualization. **Michiko Nakazato:** Writing – review & editing, Methodology. **Jinko Yokota:** Writing – review & editing, Methodology. **Kaori Koga:** Writing – review & editing, Supervision, Funding acquisition.

## Declaration of generative AI and AI-assisted technologies in the writing process

During the preparation of this work the authors used DeepL in order to improve language and readability. After using this tool, the authors reviewed and edited the content as needed and take full responsibility for the content of the published article.

## Funding source

This study was funded by the Ministry of Health, Labour and Welfare of Japan (grant number: 23FB0301). The institution played no role in designing the study, analyzing or interpreting the data, or deciding to submit the manuscript.

## Declaration of competing interest

The authors declare that they have no known competing financial interests or personal relationships that could have appeared to influence the work reported in this paper.

## Data Availability

Data will be made available on request.
